# The Challenges Imposed by Dengue, Zika, and Chikungunya to Brazil

**DOI:** 10.3389/fimmu.2018.01964

**Published:** 2018-08-28

**Authors:** Paolo Marinho de Andrade Zanotto, Luciana Cezar de Cerqueira Leite

**Affiliations:** ^1^Laboratory of Molecular Evolution and Bioinformatics, Department of Microbiology, University of São Paulo, São Paulo, Brazil; ^2^Laboratório de Desenvolvimento de Vacinas, Instituto Butantan, São Paulo, Brazil

**Keywords:** emerging infectious diseases, Dengue virus, Zika virus, Chikungunya virus, Brazil

## Abstract

Brazil has a well-established immunization program in which vaccines are provided through the Public Health System free of charge to the whole population, obtaining high coverage and reducing the incidence of important infectious diseases in children and adults. However, the environmental changes and high mobility rates of the population occurring in the last decades have triggered the sequential introduction of a series of vector-borne emerging infectious diseases, such as Dengue, Zika, and Chikungunya, that have imposed a considerable burden on the population, with yet unmet solutions. The first to be introduced in Brazil was the Dengue virus, reaching epidemic levels in 2010, with over 1 million cases annually, maintaining high infection rates until 2016. Brazil has invested in vaccine development. The Zika virus infection, initially assumed to have appeared during the World Cup in 2014, was later shown to have arrived earlier in 2013. Its emergence mobilized the Brazilian scientific community to define priorities and strategies, that rapidly investigated mechanisms of pathogenesis, differential diagnostics, and determined that Zika virus infection *per se* causes relatively mild symptoms, however, in pregnant women can cause microcephaly in the newborns. The diagnostics of Zika infection is confusing given its similar symptoms and cross-reactivity with Dengue, which also hindered the appraisal of the extent of the epidemics, which peaked in 2015 and finished in 2016. Another complicating factor was the overlap with Chikungunya virus infection, which arrived in Brazil in 2014, being prevalent in the same regions, with similar symptoms to both Dengue and Zika. Although Dengue infection can be fatal and Zika infection in pregnant woman can lead to newborns with microcephaly or an array of neurodegenerative manifestations, the Chikungunya infection is a debilitating disease leaving chronic sequelae, which unfortunately has received less attention. Precise differential diagnostics of Dengue, Zika, and Chikungunya will be necessary to evaluate the actual extent of each of these diseases during this overlapping period. Here we review the impact of these emerging infections on public health and how the scientific community was mobilized to deal with them in Brazil.

## Introduction

Brazil has a well-established immunization program in which vaccines are provided through the Public Health System free of charge to the whole population, obtaining high coverage, and reducing the incidence of important infectious diseases in children and adults.

However, despite the efforts to reduce the burden of vaccine-preventable diseases, the environmental changes and high mobility rates of the population occurring in the last decades have triggered the sequential introduction of a series of vector-borne emerging infectious diseases, such as those caused by Dengue virus (DENV), Zika virus (ZIKV), and Chikungunya virus (CHIKV), and more recently Yellow fever virus (YFV), that have imposed a considerable burden on the population, with yet unmet solutions. These emerging infectious diseases can remain at reduced levels for varying periods, reaching epidemic levels during outbreaks and are cyclic in nature, depending on the presence of the *Aedes aegypti* vectors.

## The arbovirus

**Figure 1:**
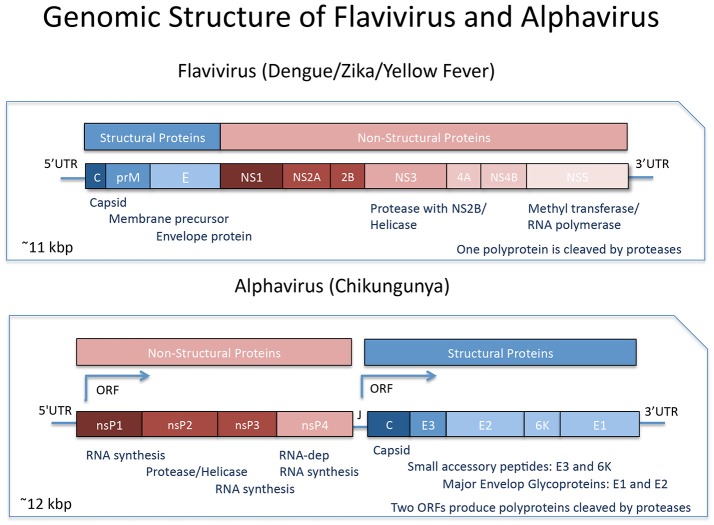
Genomic Organization of Flavivirus and Alphavirus. Flavivirus comprise a single Open Reading Frame with the genes for the Structural Proteins followed by the Non-Structural Proteins transcribed and translated and the resulting polyprotein undergoes proteolytic processing. Alphavirusus are comprised of the genes for the Non-Structural Proteins followed by the Structural Proteins, transcribed from two distinct ORFs, and each resulting polyprotein undergoes further proteolytical processing. Modified from Shi and Gao (2) and Powers et al. (3).

Although all these viruses share a common set of urban vectors, *Aedes aegypti*, and *albopictus* worldwide, they have distinct genomes and evolutionary histories. The genus *Flavivirus* (family *Flaviviridae*), such as the DENV, ZIKV and also YFV, are transmitted by arthropods such as, mosquitoes, ticks, mites, etc. (1). These viruses have a capsid with icosahedral symmetry spherical, surrounded by a viral envelope made of a lipid bilayer, with approximately 50 nanometers (nm) in diameter (1) The *Flavivirus* genome consists of a linear RNA molecule with positive polarity (+ ssRNA) of approximately 11 kilobases (Kb) in length, with a single open reading region (RLA), which encodes a polypeptide of approximately 3,400 amino acids flanked by two non-coding regions (5′ and 3′ UTR) at the ends of the genome (Figure 1). The first three proteins are the structural proteins (capsid-C, membrane precursor-prM, and envelope-E), and the last seven are non – structural proteins (NS1-NS5) (1, 4).

On the other hand, the genus *Alphavirus* and *Rubivirus*, both belong to the *Togiviridae* family. The genus *Rubivirus* includes only one species of virus, the Rubella virus, whereas the *Alphavirus* constitute a group of viruses with a more diversified molecular and antigenic classification (3, 5). Viruses belonging to the *Alphavirus* genus are often classified as New or Old World *Alphaviruses*, according to the geographical location in which they were originally isolated (6). Among the New World *Alphaviruses* there are several viruses which typically cause encephalitis in humans and other mammals, while Old World *Alphaviruses*, include viruses that cause fever, rash, arthralgia and rarely cause lethality, such as the Chikungunya virus (CHIKV), Semliki forest virus (SFV), and others (5). *Alphaviruses* also have a + ssRNA approximately 12 Kb in length, but they constitute a completely different group when compared to *Flavivirus* in terms of molecular architecture, although both have small icosahedral, enveloped capsids. The genome of viruses belonging to this genus is organized into two distinct RLAs (7), with genes located in the first RLA responsible for the synthesis of non-structural proteins and those located in the second RLA responsible for the synthesis of structural proteins (Figure 1).

## A brief account on their epidemiology

In the last three decades, DENV was the arbovirus that caused the greatest public health problems in Brazil, with continuous reintroductions that were responsible for the maintenance of the virus in the country and the introduction of new lineages (8). CHIKV was first documented in Brazil in 2014 (9), followed by the ZIKV in 2015 (10). Since then, Brazil has been experiencing the co-circulation of DENV, ZIKV, and CHIKV viruses with hyperendemic (i.e., concomitant) circulation of the four DENV serotypes (8, 11).

The first dengue epidemic reported in Brazil was in 1845, followed by outbreaks in the 1850's and 1920's. The eradication of *Aedes aegypti* in the 1930s through a Program coordinated by PAHO maintained it away until the vector was reintroduced in 1976 from the Caribean. All four serotypes of DENV were systematically reintroduced in Brazil from the Caribean in the 1980s. Initially DENV-1 and DENV-4 in the northern region, followed by a larger outbreak in Rio de Janeiro of DENV-1 in the late 1980's and another in the northeast region in the 1990's (12). It has been suggested that DENV-1 and DENV-4 cause milder disease symptoms than DENV-2 and DENV-3, and the first outbreaks of DENV-2 occurred in the 1990's with increasing cases of severe dengue (SD) and consequent fatalities. DENV-3 was introduced in the 2000s and became the most prevalent serotype, after which it alternated with DENV-2 causing high incidence of SD, spreading to several states and regions. All serotypes of DENV circulated in alternated fashion in distinct localities in Brazil with a pattern of increasing prevalence in time. Epidemic levels occurred since 2010, and eventually in 2013, all four serotypes reached a hyperendemicity status as shown in the State of São Paulo (8). There were over 1 million cases annually, maintaining high infection rates until 2016. Nevertheless, the processes that shape the transmission patterns at urban scales for these emergent viruses are poorly understood, especially the impact of factors such as human population movement and urbanization, all of which are crucial for optimal vaccine development, vaccination strategies, and public health intervention planning.

The Dengue epidemic was a public health concern during the World Cup in 2014 due to the high transmission rates and the mass gatherings occurring when Brazil hosted about 600,000 foreign visitors. Nonetheless, the quantitative risk was considered to be low due to previous exposure of the host population to circulating virus (13). The strategies currently used to contain DENV infections rely on vector control, such as public awareness campaigns, use of insecticides and vector monitoring systems. Nevertheless, new strategies interfering either with the virus infectivity (*Wolbachia* bacteria) or vector viability (Transgenic mosquitos—Oxitec) are being developed and tested (12).

As a consequence of the re-circulation of the *Aedes aegypti* vector, other emergent viruses were introduced through different paths. For example, the ECSA genotype of CHIKV came directly into Brazil from Africa, with an outbreak in Bahia, while the Asian CHIKV genotype came from Haiti into the northern region (9). CHIKV entered Brazil in 2014 with a rapid and explosive spread leading the Pan American Health Organization (PAHO) and the Centers for Disease Control and Prevention (CDC) to issue a guide to prevent future CHIKV epidemics in the Americas (14).

The ZIKV infection was first reported in 2015, initially assumed to have arrived during the World Cup in 2014, but later shown to have arrived earlier in 2013 (10, 15). ZIKV was probably introduced from the Pacific (French Polynesia or Easter Island). An outbreak of exanthematous illness was initially associated with DENV and CHIKV in Salvador, Bahia, latter identified as the ZIKV (11, 16). The ZIKV infection was mistakenly diagnosed by its similar symptoms and cross-reactivity with DENV, which also hindered the evaluation of the extent of the epidemics. Another complicating factor was the overlap of ZIKV and CHIKV infections, being prevalent in the same regions, with similar disease presentation (17, 18). Similar co-circulation of ZIKV and CHIKV was previously observed in the Pacific (19, 20). The rapid spread of ZIKV was also a concern for the high population gatherings during the 2016 Olympic Games, which brought together millions of international visitors at risk of further disseminating the outbreak (21). However, the epidemic peaked in 2015 and was finished by 2016.

## Diseases and sequelae

DENV infections can display varying outcomes, from asymptomatic to relatively mild flu-like symptoms, up to severe dengue leading to a significant proportion of case fatalities (22). On the other hand, CHIKV and ZIKV have emerged worldwide as true highly pathogenic viral pathogens for humans (14, 23). They have experienced significant geographical expansions, which in less than 10 years led to the crossing of the Pacific Ocean, reaching the American continent (9, 15). Although the ZIKV infection *per se* causes relatively mild symptoms, by the end of 2015, the physician Adriana Melo, reported the potential association between microcephaly cases and ZIKV infection during gestation and Celina Turchi coordinated the task-force that established the evidences that confirmed an association.

In November 2015, the Brazilian Ministry of Health (MS) declared a state of national public health emergency because of the ZIKV outbreak, with the objective to provide greater impulse and agility to the investigations. With the continuing increase of the epidemic, in February 1st, 2016 the World Health Organization declared that the ZIKV epidemic was a global public health emergency (http://www.who.int/emergencies/zika-virus/en/). FAPESP, a research funding institution from São Paulo, established a fast-track for Zika projects. These measures demonstrate the high concern of these governmental entities with this epidemic, which constituted a serious public health threat, with potentially immense economic and social importance. This situation mobilized the Brazilian scientific community to define priorities and strategies that rapidly investigated mechanisms of pathogenesis and differential diagnostics methodologies. Soon, Cugola et al. (23) provided the causal proof of the association between ZIKV and microcephaly by using *in vivo* and *in vitro* systems. More recently, a mouse model of ZIKV teratogeny with early embryo exposure to the virus reproduced the severe malformations and delayed development of the embryos (24). The study of ZIKV infection in discordant twins has brought insights into the role of the susceptibility of neural progenitor cells (25).

Although Dengue infection can be fatal and ZIKV infection in pregnant women can lead to microcephaly in the infant, CHIKV infection is a debilitating disease, leaving chronic sequelae, which unfortunately has received less than necessary attention. A precise differential diagnostics of Dengue, Zika, and Chikungunya at the point of action will be necessary for a much needed evaluation of the actual extent of each of these diseases during this overlapping period (26).

## Vaccine development, preparedness and resistance to vaccination

Although the incidence of DENV and ZIKV in Brazil has decreased in 2017, it is still important to develop vaccines for these diseases due to the cyclic nature of the epidemics and its possible spread to other locations. Estimation of the dengue hospitalization costs in Brazil and recent vaccine efficacy trials (27), set the stage for determining the cost effectiveness of new dengue vaccines, even considering their low efficacy levels, once incorporating the effect of herd immunity (28).

There are a few vaccines in development against DENV that have reached clinical trials. The first is a live attenuated tetravalent vaccine composed of the pre-membrane and envelop proteins of DENV of each serotype with the non-structural and capsid proteins of the attenuated yellow fever vaccine virus YF-17D developed by Sanofi. This vaccine has undergone a Phase III trial in Asian-Pacific and Latin American countries, showing efficacy between 47 and 83%, depending on the serotype, higher for children older than 9 years (66%) than for those lower than 9 years (45%) (26, 29), and has been registered for commercialization. However, post-marketing studies have recently determined that this vaccine can increase the risk of severe dengue in individuals susceptible to infection with DENV (i.e., not-previously infected) (30).

Brazil has invested in the development of live attenuated dengue vaccines (31, 32), one of which results from a collaboration between the US National Institutes of Health and Instituto Butantan. Estimated cost of production for this vaccine concluded that it would be affordable for most developing countries (31). This is a tetravalent live attenuated vaccine currently in a multi-center Phase III clinical trial (33). Another live attenuated vaccine is a chimeric construct based on DENV-2 backbone, developed by Takeda (Japan), which will be entering clinical trials (33).

Following the emergence of the ZIKV outbreak in Brazil and its association with microcephaly, a global effort for the development of vaccines was launched, stimulated by WHO's declaration of a public health emergency. The first strategies pursued which showed protection against ZIKV challenge in mice were DNA vaccines and inactivated virus vaccines due to the advantages of these platforms in terms of quick development (34, 35) and these have progressed rapidly (36). Mid 2016, WHO and UNICEF organized a working group for consultation in the development of a ZIKV vaccine Target Product Profile for use in a future emergency outbreak, laying out guidelines for developers and regulators (37). At that time there were over 30 vaccine candidates in development using a large variety of different strategies, including mRNA vaccination or Virus-Like Particles based vaccines, with promising results (38–40) and the more advanced ones had undergone FDA approval for clinical trials. The discussions raised a series of points on the best pathways forward concerning safety and regulatory issues (41). Although the local and global efforts lead to early developments of vaccine candidates, the decline in cases and unforeseen emergent outbreaks may hinder further progress in their development (42).

On the other hand, the decline in ZIKV incidence was closely followed by a devastating outbreak of Yellow Fever (YF) in nonhuman primates, initiating in Minas Gerais, early in 2017, and spreading to Espírito Santo, São Paulo, and Goiás (43). Because of significant spillover into the human population, this alarming outbreak triggered mobilization of public health measures to contain the spread of the wild type (jungle) YF and hinder the onset of urban YF (44). Mass vaccination campaigns were initiated to cope with the increasing number of reported and confirmed human cases. Two important factors took place in dealing with this outbreak. Once the first fatalities due to YF were announced, public alarm initially triggered a rush to the public health system in search for immunization. The sudden increase in demand for vaccine resulted in shortage of vaccine stocks and enormous lines formed by the population at immunization sites. Since the production of vaccine is a long process, the decision was toward vaccine fractionation, previously demonstrated to be efficacious. WHO sent a small emergency stockpile and Biomanguinhos, Fiocruz, expedited vaccine production to meet the plan to achieve the immunization of 20 million individuals in endemic areas.

On the other hand, we believe that mass vaccination can increase the otherwise small level of adverse events occurring due to the vaccine. At the same time the media overreacted prematurely amplifying through social networks concerns on the adverse effect of the vaccine, which found resonance in the incipient but increasing anti-vaccine movement. As a result of general perception, vaccine resistance became an important factor in this outbreak.

On a whole, it is clear that the presence of the vector has facilitated consecutive virus outbreaks and it will be important to invest more efficiently in vector control. On the other hand, while it is still around, close surveillance has identified early signs of different outbreaks of emerging infections, which has been essential to allow prompt organization of public health measures. The scientific community and government sectors have been mobilized toward the investigation of the different pathogens, bringing insights into their epidemiology and pathogenesis, new vaccine developments or increasing vaccine supply, depending on their respective state of knowledge and development. Considering the severity of these diseases, we will always consider that the whole process can be expedited and improved in order to reduce the burden on public health.

## Author contributions

PZ and LL wrote sections of the review. Both authors contributed to manuscript revision, read and approved the submitted version.

### Conflict of interest statement

The authors declare that the research was conducted in the absence of any commercial or financial relationships that could be construed as a potential conflict of interest.
